# *CSN1S1* and *LALBA* Polymorphisms and Other Factors Influencing Yield, Composition, Somatic Cell Score, and Technological Properties of Cow’s Milk

**DOI:** 10.3390/ani13132079

**Published:** 2023-06-23

**Authors:** Jindřich Čítek, Eva Samková, Michaela Brzáková, Oto Hanuš, Libor Večerek, Irena Hoštičková, Eva Jozová, Lucie Hasoňová, Karolína Hálová

**Affiliations:** 1 Department of Genetics and Agricultural Biotechnology, Faculty of Agriculture, University of SouthBohemia in České Budějovice, Studentská 1668, 370 05 České Budějovice, Czech Republic; vecerek@fzt.jcu.cz (L.V.); hostickova@fzt.jcu.cz (I.H.); jozovae@fzt.jcu.cz (E.J.); 2 Department of Food Biotechnologies and Agricultural Products Quality, Faculty of Agriculture, University of South Bohemia in České Budějovice, Studentská 1668, 370 05 České Budějovice, Czech Republic; samkova@fzt.jcu.cz (E.S.); hasonova@fzt.jcu.cz (L.H.); halovk03@fzt.jcu.cz (K.H.); 3 Institute of Animal Science, Přátelství 815, 104 00 Praha-Uhříněves, Czech Republic; brzakova.michaela@vuzv.cz; 4 Dairy Research Institute, s.r.o., Ke Dvoru 12a, 160 00 Prague 6, Czech Republic; hanus.oto@seznam.cz

**Keywords:** simmental, holstein, milk performance, milk quality, somatic cell score

## Abstract

**Simple Summary:**

Animal breeding is one of the most profitable human activities. The ultimate goal is to join genetically high performance and good health. In the last decades, the study of genetic control of important traits on the molecular level has been launched. In the study of milk performance, major genes, namely those for milk proteins, are the focus of much research. Here, we studied the polymorphisms of *CSN1S1* and *LALBA* genes and their associations with milk performance and quality. Also, other non-genetic effects were evaluated. The polymorphisms influenced milk, fat, and protein yield percentages in the second lactation but their effects in the first lactation were not significant. The technological properties of milk were not influenced. The effect of the farm was significant in most cases. Breed influenced milk performance but did not influence technological properties. Changes in lactose content reflect the health of the udder.

**Abstract:**

We evaluated the influence of *CSN1S1* and *LALBA* polymorphisms on cow’s milk yield and quality. The analysis was done on Czech Simmental and Holstein cows. Non-genetic factors were included as well. *CSN1S1* did not influence the milk performance in the first lactation. In the second lactation, cows with the *BB* genotype had significantly higher milk, protein, and fat yields than *BC*. The differences between *LALBA* genotypes were non-significant in the first lactation, while in the second lactation, the fat percentage was significantly higher in *BB* than in *AB*. The farm significantly influenced milk, protein, and fat yields in both the first and second lactations and fat percentage in the first lactation. The effect of *CSN1S1* and *LALBA* genotypes on the milk technological quality was non-significant. Breed did not influence any of the evaluated technological traits and SCS. The ethanol test was not influenced by farm, season, lactation phase, protein percentage, breed, or non-fat solids percentage. Farm, season, and protein percentage significantly influenced milk fermentation ability, renneting, and SCS. The lactose content is a good indicator of udder health.

## 1. Introduction

Cattle breeding is an important and profitable branch of livestock production and represents an indicator of the development of the agricultural and food sector as a whole. The harmonization of livestock with field production contributes to achieving greater stability of production on the farm and, in general, the overall agriculture of the country [1]. Today’s dairy cattle breeding faces several challenges that include the sustainability of livestock production and its impact on climate change, limited sources of feed and water, and growing public interest in animal welfare and the quality of animal products. For more than twenty years, the research has focused on functional traits like longevity, fertility, and health, which have deteriorated due to long-term selection for high milk production [2]. Therefore, the approach of breeders must be all-encompassing. For dairy farms, it is important that milk yield and composition markedly influence their economy. For processing plants, technological properties, in addition to composition, are important; therefore, different effects on both characteristics are studied [3,4,5,6,7]. Concerning the genetic background of technological properties, low heritabilities are found [6,8]. Others report the heritabilities of nine cheese-making properties between 0.37 and 0.48 [9].

Though omics technologies are used as of late [10], the effects of individual major gene polymorphisms are still being studied. Exhaustive surveys of cow´s milk proteins, including the content in milk, genetic variants, and their sequences, positions, and amino acid differences, are available [11,12]. Other authors have focused only on caseins [13]. 

The CSN1S1 family (alpha-S1-casein) constitutes up to 40% of the casein fraction in cow´s milk. There are nine variants of the gene, and *B* is the most frequent [11,12]. In Pinzgau cattle, for example, the frequency of the *B* allele was 0.930, and the frequency of the *C* allele was 0.070, while the *CC* genotype was not found at all [14]. In Holstein and Swedish Red, similar frequencies were found; only in Jersey cattle was there a higher proportion of the *C* allele and *CC* genotype [7]. Ozdemir et al. [15], in a review and meta-analysis, report on the significant influence of *CSN1S1* gene polymorphism on protein, fat yield, and content, but other authors have reported that the relationships between the yield traits and the genotypes are not significant. The *BC* genotype of *CSN1S1* was found to be better in terms of milk protein content in the Holstein and Simmental breeds and in terms of milk fat content in the Simmental breed [16]. Milk yield was not significantly associated with *CSN1S1* genotypes. Other authors found a significant effect of the *CSN1S1* locus on breeding values for milk yield but not for protein and fat content and yields [17]. The best breeding values had *CC* genotypes for all milk traits, but the frequency of the genotype was low, so definite conclusions could not be drawn. Authors studying technological quality found that the *BB* genotype worsened the rennet coagulation of milk [18]. In an analysis covering five polymorphous loci, i.e., *STAT1*, *OLR1*, *CSN1S2*, *DGAT1*, and *CSN1S1*, the last mentioned was found to not significantly affect milk yield traits and the content of milk components in Holsteins cows [19].

Lactose synthase catalyzes the formation of lactose, which is the major osmole saccharide of bovine milk and regulates milk volume. Lactalbumin alpha (LALBA) is involved in the synthesis of lactose synthase in the mammary gland, facilitates milk production and secretion, binds the divalent cations Ca^2+^ and Zn^2 +^, and may facilitate the absorption of essential minerals. Therefore, *LALBA* is regarded as a plausible candidate gene for the milk yield trait [20,21]. In Holstein cows, the *BB* genotype showed a non-significantly higher 305-day milk yield than *BA*. The content of milk fat, protein, lactose, and non-fat solids was non-significantly higher in cows with the *BA* genotype, while the yields of milk components were non-significantly higher in the *BB* genotype due to the high milk yield [22]. Other studies in the same breed showed contrarily better milk yield and fat percentage in animals with the *AA* genotype [23] and found that the populations of different Norwegian breeds were monomorphic, with the *B* variant comprising 100% [24]. More polymorphous was the population in another study with the following frequencies: *LALBA AA* 59.7%, *AB* 35.7%, and *BB* 4.6% [25]. The authors found an association between *LALBA* genotypes and the levels of blood indices characterizing defense functions.

The aim of this paper is the association study of polymorphisms in *CSN1S1* and *LALBA* genes with the performance, composition, and technological qualities of cow milk. Analysis was done in field conditions on several farms. Other factors potentially affecting the performance and quality of milk were evaluated as well.

## 2. Materials and Methods

DNA was extracted non-invasively from milk samples. The research was conducted under the supervision of the Institutional Animal Care and Use Committee of the Faculty of Agriculture of South Bohemia University, where the experiment was carried out under approval number 22036/2019-MZE-18134.

### 2.1. Animals and Genotyping

In the paper, 191 cows were included; of these, 121 were Czech Simmental and crosses with a proportion of the breed over 75%, and 70 were Holstein and crosses with a proportion of the breed over 81%. The cows were kept in five farms in the Czech Republic and calved between 2015 and 2017. Milk samples were obtained repeatedly in the first and second lactations.

DNA was isolated from the milk samples using the DNA/RNA extractor MagCore HF16 Plus (RBC Bioscience) MagCore^®^ Genomic DNA Tissue Kit. Genotyping was performed using the PCR/RFLP method. The alleles *B* and *C* in the *CNS1S1* gene were genotyped as in Soyudal et al. [22]. The primer sequences were F: 5′ -TTGGTTTTACTGGCCTCTCTTGTCATC- 3′ and R: 5′ -TGAATTATGGGACAAAGCA AAATAGCAG- 3′. The PCR reaction was carried out in a volume of 25 µL,1x reaction buffer PPP Master Mix (Top-Bio, CZ) (75 mM Tris-HCl, pH 8.8, 20 mM (NH_4_)_2_SO_4_, 0.01% Tween 20, 2.5 mM MgCl_2_, 200 µM each dNTP, 50U/mL Taq DNA polymerase), 10 pmol of each primer, 50 ng of template DNA, 7.5 µL H_2_O. Microtubes with solution were placed in a thermal cycler TProfessional Basic Gradient (BiometraGmbH, Göttingen, Germany) with the following program: 1 cycle 95 °C for 5 min, 30 cycles 95 °C for 40 sec, 58 °C for 60 s, 72 °C for 90 s, and final elongation at 72 °C for 10 min. The alleles were distinguished by splitting with *Msp*I endonuclease. 

The alleles *A* and *B* in the *LALBA* gene were genotyped as in Lien et al. [26]. The primer sequences were F: 5′ -ACAATTCTACCAGCTGGATGCCTATC- 3′ and R: 5′ -CACGCTCCACAGTTCCTGAGTAA- 3′ for outer primers and R: 5′ -CACGCTCCACAGTTCCTGAGTAA- 3′ and F: 5′ -CCGGATCCTATTGGCTCTGAGAACGGTG- 3′ for inner primers. The reaction mixture was the same. The temperature program was: 1 cycle 95 °C for 5 min, 30 cycles 95 °C for 15 s, 60 °C for 30 s, 72 °C for 30 s, and final elongation at 72 °C for 10 min. The alleles were distinguished by *Hph*I. Products PCR-RFLP were visualized on 2.5% agarose gel.

### 2.2. Milk Performance and Quality

Data on milk performance were collected from the milk breeders’ databases of records. Milk yield in kg, fat, and crude protein percentages was evaluated. The milk composition, i.e., the content of fat, crude protein, casein, lactose monohydrate, non-fat solids (NFS), urea, citric acid, acetone, ketone as beta-hydroxybutyrate (BHB), and somatic cell count, was determined in breeder milk laboratories of the Czech–Moravia Breeders Association using infrared spectroscopy in the mid wavelength area by filter technology and by such technology as Michelson’s interferometer and Fourier’s data transformation (FT), Foss Electric (Hillerød, Denmark) and Bentley Instruments (Chaska, MN, USA) instrumentation (Combi Foss 6000 and Bentley 2500 and Somacount). The milk instruments used to determine milk composition were calibrated monthly for the main milk components (fat, crude protein, and lactose monohydrate) according to the results of the relevant reference methods (Kjeldahl mineralization, distillation, and titration method for crude protein as milk total nitrogen × 6.38 and the Roese–Gottlieb extraction and gravimetric method for milk fat, enzymatic determination of lactose on a spectrophotometer absorbance at 340 nm with the commercial Lactose/D-Galactose-Assay Kit from MEGAZYME [27]. The laboratories are accredited for official milk recording analysis in the Czech Republic according to ICAR (International Committee for Animal Recording) and by the Czech Accreditation Institute according to the valid international standard [28].

Technological properties were evaluated by milk fermentation ability (titration acidity of yogurt test). Renneting was measured subjectively and instrumentally, and the heat stability of the milk (ethanol or alcohol test) was determined. Technological properties were repeatedly measured during the first and second lactations.

The heat stability of milk was measured indirectly using milk ethanol stability, determined by milk titration (5 mL) with 96% ethanol to the point of the creation of the first visible milk protein precipitated flakes and is reported as mL of alcohol.

The milk fermentation ability or the yogurt test was carried out according to the Czech milk industry-standard ON 57 0534 [29]. The sample of raw milk (50 mL) was heated at 85 °C for 5 min and cooled at 43 ± 2 °C. Subsequently, the sample was inoculated with 2 mL of the thermophilic lactic culture YC-180-40-FLEX (Chr. Hansen, Denmark; *Streptococcus thermophilus*, *Lactobacillus delbrueckii* subsp. *lactis*, and *L. d.* subsp. *bulgaricus*). The inoculated sample was incubated at 43 °C for 3.5 h. The result was expressed as the titration acidity of the yogurt in mL of 0.25 mol × L^−1^ NaOH × 100 mL^−1^ (or the so-called degree according to Soxhlet–Henkel).

Rennetability (classical procedure) was determined during tempering (35 °C) of a defined milk volume after the addition of the rennet (1% vol.; bacterial rennet matter, enzyme Fromase 75TL), usually by measuring the time, while held at 35 ± 0.5 °C (rennet coagulation time, RCT, for milk protein) until the occurrence of the first flakes of lactoproteins (beginning of coagulation).

Rennetability was also determined using nephelometry (turbidimetry measurement) for the assessment of the milk coagulation time (ML-2 analyzer, manual operation). This is the use of the optical method (NEF) for evaluating the intensity of the so-called diffusely scattered Tyndall light on dispersed particles (coagulating lactoprotein flakes). 

### 2.3. Statistical Analysis

The association analysis between gene polymorphism and milk production and quality was performed using the mixed linear model (MIXED procedure with repeated measurements) based on the least-squares method in Statistical Analysis Software v. 9.4 (SAS 9.3, SAS Institute, Cary, NC, USA). The models for the evaluation of the association between evaluated traits and genotypes of *CSN1S1* and *LALBA* genes were developed as follows:

(a) For milk yield and composition:
  Y_jlk_ = gen_i_ + farm_j_ + breed_k_ + sire_l_ + e_ijkl_, (1)
  where Y_jlk_ is the analyzed trait (milk yield, milk fat, and protein in % and kg); gen_i_ is the fixed class effect of genotype (i = 1, 2) for the *CSN1S1* or *LALBA* gene; farm_j_ is the fixed class effect of farm (j = 1, 2, 3, 4, 5); breed_k_ is the fixed class effect of breed (Holstein or Czech Simmental); sire_l_ is the random effect of sire; and e_ijkl_ is a random residual.
(b) For milk quality:
  Yogurt_ijklmno_ = gen_i_ + farm_j_ + lacs_k_ + season_l_ + protein_m_ + pe_n_ + sire_o_ + e_ijklmno_, (2)
  where Yogurt_ijklmno_ is the yogurt test values; gen_i_ is the fixed class effect of genotype (i = 1, 2) for the *CSN1S1* or *LALBA* gene; farm_j_ is the fixed class effect of farm (j = 1, 2, 3, 4, 5); lacs_k_ is the fixed class effect of the lactation stage in days (k = 1, 2, 3); season_l_ is the fixed class effect of the season (l = 1, 2, 3, 4); protein_m_ is the fixed effect of protein % in milk; pe_n_ is the permanent effect of the cow; sire_o_ is the random effect of sire; and e_ijklmno_ is a random residual.
(c) For rennetability:
  Rennetability_ijklmno_ = gen_i_ + farm_j_ + season_k_ + protein_l_ + NFS_m_ + pe_n_ + sire_o_ + e_ijklmno_, (3)
  where Rennetability_ijklmno_ is the rennetability assessed subjectively or instrumentally; gen_i_ is the fixed class effect of genotype (i = 1, 2) for the *CSN1S1* or *LALBA* gene; farm_j_ is the fixed class effect of farm (j = 1, 2, 3, 4, 5); season_k_ is the fixed class effect of the season (k = 1, 2, 3, 4); protein_l_ is the fixed effect of protein % in milk; NFS_m_ is the fixed effect of non-fat solids % in milk; pe_n_ is the permanent effect of the cow; sire_o_ is the random effect of sire; and e_ijklmno_ is a random residual.
  Ethanol_ijkl_ = gen_i_ + farm_j_ + pe_k_ + sire_l_ + e_ijkl_, (4)
  where Ethanol_ijkl_ is the ethanol stability; gen_i_ is the fixed class effect of genotype (i = 1, 2) for the *CSN1S1* or *LALBA* gene; farm_j_ is the fixed class effect of the farm (j = 1, 2, 3, 4, 5); pe_k_ is the permanent effect of the cow; sire_l_ is the random effect of sire; and e_ijkl_ is a random residual.
(d) For SCS:
  SCS_ijklmn_ = gen_i_ + farm_j_ + lacs_k_ + month_l_ + pe_m_ + sire_n_ + e_ijklmn_, (5)
  where SCS_ijklmn_ is the somatic cell score; gen_i_ is the fixed class effect of genotype (i = 1, 2) for the *CSN1S1* or *LALBA* gene; farm_j_ is the fixed class effect of farm (j = 1, 2, 3, 4, 5); lacs_k_ is the fixed class effect of the lactation stage in days (k = 1, 2, 3); month_l_ is the fixed class effect of the month (l = 1, …, 11); pe_m_ is the permanent effect of the cow; sire_n_ is the random effect of sire; and e_ijklmn_ is a random residual.

Milk yield and composition were evaluated separately for the first (n = 170) and second (n = 156) lactations. The number of records included in the milk performance evaluation is shown in Table S1. Milk technological quality traits were recorded repeatedly, and the number of records ranged from one to five per cow. The permanent environment effect was included in the model due to repeated measurements. Approximately 40% of cows had only one record, 35% had two records, 20% had three records, and 5% reached four or five records during the first two lactations. However, the statistically significant differences between lactation parities were not observed, so the lactation parity effect in the models was not included. The lactation stage (the number of days milking per cow per lactation) was divided into early (1 to 99 days), mid (100 to 200 days), and late (201 to 305 days) lactation stages. The season effect was created by merging three consecutive months of the year into four groups (1-December, January, February; 2-March, April, May; 3-June, July, August; 4-September, October, November) to reflect the changing environmental conditions during the year.

The number of somatic cell counts was converted to a log score (somatic cell score) to ensure the normal distribution of the trait according to the following formula:

SCS = log2 (SCC/100) + 3,(6)

where SCS is somatic cell score, and SCC is the number of somatic cell counts (in thousands of units). Only SCC in the range of 13 to 9999 was considered in the analysis. 

The analyzed dataset was small. For analysis simplicity, the pedigree of the cows was not included. The connectivity between animals (contemporary groups) was ensured by the random sire effect. Each sire had to have at least two daughters. On average, there were 5 daughters per sire, and the maximum was 27 daughters. 

The phenotype correlation between chosen traits was computed by Pearson correlation coefficients (CORR Procedure, SAS 9.4). For post hoc comparisons, the Tukey–Kramer test was used [30].

Descriptive statistics of milk performance and quality are given in Tables S1 and S2.

## 3. Results and Discussion

As shown in Table 1, the frequencies of genotypes are quite in agreement with the literature when B and C are common in the *CSN1S1* gene and A and B in the *LALBA* gene [11]. In our group of cows, in the *CSN1S1* gene, allele *B* has a frequency of 0.937 and allele *C* of 0.063. Genotype *CC* was not present. In the *LALBA* gene, allele *A* has a frequency of 0.365 and allele *B* of 0.635. Genotype *AA* was not present. This agrees with most authors, as mentioned below [11,31,32,33].

As given in Table 2, the *CSN1S1* genotypes *BB* and *BC* did not influence the milk yield, protein and fat percentages, and yields in the first lactation. In the second lactation, cows with the *BB* genotype had a significantly higher milk yield, which resulted in significantly higher protein and fat yields when the percentages were the same in both genotypes. This confirmed the trend that was also apparent in the first lactation, in which the differences were non-significant. Thus, the *BB* genotype seems to be slightly superior. However, the number of cows of the *BC* genotype was relatively low, which could influence the results. These results are basically in agreement with Ozdemir et al. [15]. Other authors refer to better milk yield breeding values for the *BB* genotype but not for protein and fat yields, as in this paper [17]. Kim et al. [34] and Ketto et al. [35] did not find significant associations between *CSN1S1* genotypes and milk performance or milk protein content. However, others refer to the higher protein and casein percentages in the *BB* genotype compared to the *BC* genotype [36]. Tyulkin et al. [23] refer to the better performance for the *BC* genotype.

For *LALBA* genotypes, the differences were non-significant in the first lactation, but for protein and fat percentages, the *p*-values were near the significance threshold. In the second lactation, the protein percentage was near the significance threshold as well, and the fat percentage was significant at *p* < 0.05. Higher content was found in *BB* genotypes in both the first and second lactations. The differences between genotypes in protein and fat yields were low and non-significant. It is rather difficult to discuss the results with other authors, as the frequencies of the *A* allele and *AA* genotype are very low or completely missing [11,31,32,33]. However, some authors found a higher milk yield for the *BB* genotype near the significance threshold, non-significantly higher fat, protein, lactose, and milk solid content for the *AB* genotype, and non-significant yields of fat and protein higher for the *BB* genotype [22]. Tyulkin et al. [23] report a better milk yield for the *AA* genotype compared to *AB* and *BB,* but the differences in milk fat percentage were minimal.

Also, other effects on milk performance were tested (Table 3). The farm significantly influenced milk, protein, and fat yields in both the first and second lactations and fat percentage in the first lactation. The effect of the farm on the protein percentage and fat percentage in the second lactation was weak. Breed significantly influenced milk, protein, and fat yields in the first lactation, while the effects in the second lactation were non-significant except for protein percentage in the model with the *LALBA* genotypes.

The effect of the *CSN1S1* and *LALBA* genotypes on milk fermentation ability (yogurt test), renneting assessed subjectively and instrumentally, ethanol test, and somatic cell score was non-significant (Table 4). However, others refer that the *CSN1S1* C allele positively affected milk coagulation [37]. In poorly coagulating and noncoagulating milk samples, the B variant predominated [38]. Cechinato et al. [8] found an association between *CSN1S1* polymorphisms with curd firmness. Other analyses found a significant effect on the rennet coagulation properties or syneresis [18,39]. In another study, the latter authors did not find a significant effect of *CSN1S1* polymorphism on rennet coagulation properties in skim milk and milk after ultrafiltration [35]. Proteins in milk are strongly genetically associated with coagulation traits [9].

The content and composition of milk protein fractions affect the coagulation properties of milk [18,40,41,42]. Higher content of protein, casein, and other protein fractions shortened the rennet time and improved the curd. A lower proportion of CSN1S1, casein beta (CSN2), and a higher proportion of casein kappa (CSN3) in total milk casein resulted in firmer curd [40]. Other authors came to similar conclusions [4]. By contrast, the alpha S2 casein (CSN1S2) prolonged the rennet time, and CSN2 worsened the curd firmness. According to Cipolat-Gotet et al. [43], these relations also influenced the cheese yield, but this was not found for LALBA.

No significant correlations were found among protein fraction content and coagulation characteristics except for CSN2 (-0.34 for rennet time, +0.30 for curd firmness) [18]. Low correlations of 0–0.24 were also found [42]. A non-significant positive correlation was found between protein content and milk fermentation ability (yogurt test, +0.04), while a non-significant negative correlation was found between protein and rennet coagulation time (−0.06) [44]. However, the correlation with thermostability (alcohol test) was significant (−0,15; *p* < 0,05). Based on the effects of genetic variants of milk proteins on technological properties, it would be possible to use the milk of cows with specific genotypes for a particular purpose [45], though others assume that choosing optimal variants would be difficult [18,46].

For other effects evaluated in our analysis, the ethanol test was not influenced by farm, season, lactation phase, protein percentage, breed, or non-fat solids (NFS) percentage (Table 5). Breed, i.e., Holstein and Czech Simmental, did not influence any of the evaluated technological traits and SCS. Farm, season, and protein percentage significantly influenced milk fermentation ability, renneting, and SCS. The lactation stage (days in milk) influenced milk fermentation ability and SCS; non-fat solids influenced renneting. Hanuš et al. [47] carried out, within three years, an analysis of 2634 raw milk samples. The thermostability was, on average, 18.8 min and was affected by all 12 evaluated factors, e.g., year, season, milk yield, breed, milking, and summer grazing. Knowledge of these factors is useful for the effective manufacturing of milk.

The study included two breeds, Holstein cattle and Simmental. Despite the different phenotypes of both breeds, statistically significant differences were observed only in production traits. Holstein cattle performed higher milk production but lower milk components compared to Simmental cattle. The influence of genotype and breed interaction was also tested (data not shown). A statistically significant interaction between the *CSN1S1* gene polymorphism and breed was found during the first lactation for milk yield, protein yield, and fat yield. This finding suggests that the impact of individual polymorphisms may be influenced by the specific combination of genotypes and breeds. However, in most cases, the influence of breed alone proved statistically more significant.

Based on the renneting test and alcohol test, we can infer that Holstein cattle have lower milk quality, which may be attributed to factors such as increased microbial contamination, improper storage, or a higher incidence of mastitis in Holstein. All these factors were included in the statistical model, accounting for the farm effect.

In the end, the correlations between somatic cell score and milk constituents were computed (Table 6). Unfortunately, due to incomplete records, especially regarding milk quality traits, the number of samples varies. The correlations with milk yield and lactose percentage were negative and significant, with crude protein percentage positive and significant. Other correlations were non-significant, very low, both positive and negative. A significant negative correlation of SCS with lactose content was found by others as well [48]. It follows that lactose content is an important indicator of udder health.

## 4. Conclusions

According to our results, polymorphisms in the *CSN1S1* and *LALBA* genes affected milk yield, percentages, and yields of fat and protein in the second lactation but not in the first one. The analyzed polymorphisms did not influence the technological properties of milk. As for other effects, the farm was significant in most cases; the breed was often significant in milk performance but never in technological properties. The lactose content is a good indicator of udder health in dairy cows.

## Figures and Tables

**Table 1:** Frequencies of genotypes and alleles of Holstein and Czech Simmental cows.

Gene		Genotype	n	%	χ^2^	Allele Frequencies	
All cows	*CSN1S1*	*BB*	167	87.4	0.456 ^ns^	B	C
		*BC*	24	12.6		0.937	0.063
		*CC*	0	0			
	*LALBA*	*AA*	0	0	32.938 ***	A	B
		*AB*	103	73.0		0.365	0.635
		*BB*	38	27.0			
Simmental	*CSN1S1*	*BB*	99	81.82	49.000 ***	B	C
		*BC*	22	18.18		0.909	0.091
	*LALBA*	*AB*	67	82.72	34.679 ***	A	B
		*BB*	14	40.37		0.413	0.586
Holstein	*CSN1S1*	*BB*	68	97.14	62.229 ***	B	C
		*BC*	2	2.86		0.986	0.014
	*LALBA*	*AB*	36	60.00	2.4 ^ns^	A	B
		*BB*	24	40.00		0.300	0.700

n, number of cows with respective genotype; ^ns^ not significant, the group was in Hardy–Weinberg equilibrium; *** significant differences between genotype frequencies calculated based on Hardy–Weinberg equilibrium and empirical frequencies (*p* < 0.001).

**Table 2:** Milk yield and composition according to the *CSN1S1* and *LALBA* genotypes, LSM ± SE.

Trait	Gene and Genotype			
	*CSN1S1 ^BB^*	*CSN1S1 ^BC^*	*LALBA ^AB^*	*LALBA ^BB^*
	First lactation			
n	149	21	95	34
Milk yield (kg)	8195 ± 134	8000 ± 283	8351 ± 169	8039 ± 250
*p*-value	0.493		0.233	
Crude protein (%)	3.44 ± 0.02	3.45 ± 0.04	3.43 ± 0.03	3.51 ± 0.04
*p*-value	0.836		0.058	
Protein (kg)	279.6 ± 4.1	274.3 ± 8.8	284.4 ± 5.3	279.9 ± 7.8
*p*-value	0.551		0.580	
Fat (%)	4.14 ± 0.04	4.20 ± 0.08	4.12 ± 0.05	4.24 ± 0.07
*p*-value	0.417		0.067	
Fat (kg)	338.3 ± 5.2	336.0 ± 11.2	342.0 ± 6.9	342.7 ± 10.2
*p*-value	0.840		0.952	
	Second lactation			
n	137	19	82	35
Milk yield (kg)	9135 ± 185 ^a^	8383 ± 334 ^b^	9049 ± 223	8760 ± 295
*p*-value	0.021 *		0.337	
Crude protein (%)	3.46 ± 0.03	3.44 ± 0.05	3.45 ± 0.03	3.53 ± 0.04
*p*-value	0.780		0.085	
Protein (kg)	313.2 ± 5.9 ^A^	286.5 ± 10.0 ^B^	309.5 ± 7.0	307.3 ± 9.0
*p*-value	0.005 **		0.807	
Fat (%)	4.14 ± 0.04	4.14 ± 0.09	4.11 ± 0.05 ^a^	4.28 ± 0.07 ^b^
*p*-value	0.982		0.037 *	
Fat (kg)	375.7 ± 7.4 ^a^	346.4 ± 13.4 ^b^	369.3 ± 9.0	373.5 ± 11.8
*p*-value	0.026 *		0.722	

n, number of lactations of cows with a particular genotype; LSM, least squared mean; SE, standard error; * significant at *p* < 0.05; ** significant at *p* < 0.01; ^a,b^ different letters between genotypes in the same row represent significant differences at *p* < 0.05; ^A,B^ different letters between genotypes in the same row represent significant differences at *p* < 0.01.

**Table 3:** Significance of effects of farm and breed on milk performance, statistical models with the *CSN1S1* and *LALBA* genes.

	Farm		Breed	
	*CSN1S1*	*LALBA*	*CSN1S1*	*LALBA*
Trait	First lactation			
Milk yield (kg)	***	***	***	**
Crude protein (%)	*	ns	*	**
Protein (kg)	***	***	*	*
Fat (%)	***	**	ns	ns
Fat (kg)	***	***	**	**
	Second lactation			
Milk yield (kg)	**	***	ns	ns
Crude protein (%)	ns	ns	ns	*
Protein (kg)	***	***	ns	ns
Fat (%)	*	ns	ns	ns
Fat (kg)	***	***	ns	ns

*** significant at *p* < 0.0001; ** significant at *p* < 0.01; * significant at *p* < 0.05; ns not significant.

**Table 4:** Milk qualities according to the genotype of Holstein and Czech Simmental cows in the first and second lactations, LSM ± SE ^1^.

Trait	Gene and Genotype			
	*CSN1S1 ^BB^*	*CSN1S1 ^BC^*	*LALBA ^AB^*	*LALBA ^BB^*
Milk fermentation ability (ml NaOH)	14.78 ± 0.19	15.01 ± 0.45	14.59 ± 0.23	14.88 ± 0.37
n	290	45	185	69
*p*-value	0.620		0.469	
Renneting subjectively (sec)	497.49 ± 21.03	501.07 ± 48.87	494.27 ± 29.42	485.56 ± 53.52
n	296	42	186	69
*p*-value	0.942		0.878	
Renneting instrumentally (sec)	309.60 ± 11.62	307.97 ± 25.97	315.88 ± 14.47	289.11 ± 26.35
n	316	47	195	71
*p*-value	0.950		0.338	
Ethanol test (ml of ethanol)	0.973 ± 0.094	0.874 ± 0.173	0.946 ± 0.106	0.969 ± 0.155
n	297	46	187	72
*p*-value	0.541		0.881	
Somatic cell score	2.936 ± 0.135	3.018 ± 0.297	3.091 ± 0.192	3.042 ± 0.294
n	308	47	189	69
*p*-value	0.781		0.876	

n, number of measures of repeatedly taken samples from cows with a particular genotype; LSM, least squared mean; SE, standard error; ^1^ the effect of genotype was not significant.

**Table 5:** Significance of effects on milk quality, a model with *CSN1S1* or *LALBA* in the first and second lactations.

Trait	Model with *CSN1S1*, Effect of					
	Farm	Season	DIM ^1^	Protein (%)	Breed	NFS ^2^
Milk fermentation ability (ml NaOH)	***	***	***	***	ns	n/a
Renneting subjectively (sec)	**	*	ns	***	ns	***
Renneting instrumentally (sec)	***	*	ns	***	ns	***
Ethanol test (ml of ethanol)	ns	ns	ns	ns	ns	ns
SCS^3^	***	ns	**	*	ns	n/a
		Model with *LALBA*, effect of				
	Farm	Season	DIM ^1^	Protein (%)	Breed	NFS ^2^
Milk fermentation ability (ml NaOH)	***	***	*	**	ns	n/a
Renneting subjectively (sec)	*	**	ns	***	ns	***
Renneting instrumentally (sec)	**	ns	ns	***	ns	***
Ethanol test (ml of ethanol)	ns	ns	ns	ns	ns	ns
SCS ^3^	ns	*	ns	*	ns	n/a

^1^ Days in milk (lactation stage); ^2^ non-fat solid; ^3^ somatic cell score; *** significant at *p* < 0.0001; ** significant at *p* < 0.001; * significant at *p* < 0.05; ns not significant; n/a not available.

**Table 6:** Pearson phenotypic correlations for somatic cell score and content of milk constituents on the day of sampling.

	n	Correlation	*p*-Value
Milk yield (kg)	354	−0.187	<0.001 **
Crude protein (%)	355	0.147	0.006 **
Casein (%)	151	0.016	0.846
Fat (%)	354	0.075	0.159
Lactose (%)	355	−0.320	<0.0001 **
Citric acid (%)	354	−0.034	0.521
Acetone (mmol/L)	354	−0.058	0.280
Ketones BHB (mmol/L)	351	−0.062	0.247
Urea (mg/100 mL)	355	0.037	0.486
Non-fat solid (%)	272	0.023	0.707

** significant at *p* < 0.01; n number of samples from cows with measurements for the trait.

## Data Availability

Data are available upon request due to privacy.

## Supplementary Materials

The following supporting information can be downloaded at: https://www.mdpi.com/article/10.3390/ani13132079/s1, Table S1: Descriptive statistics of the milk performance of Holstein and Czech Simmental cows, Table S2: Descriptive statistics of the milk quality of Holstein and Czech Simmental cows.
