# Supplementary material for: CSN1S1 and LALBA Polymorphisms and Other Factors Influencing Yield, Composition, Somatic Cell Score, and Technological Properties of Cow’s Milk
Source: Animals (Basel). 2023 Jun 23;13(13):2079. doi: 10.3390/ani13132079 (PMC10339887; doi:10.3390/ani13132079)
Supplement: Supplementary file 1 [file animals-13-02079-s001.zip › animals-2421363-supplementary.pdf]

Table S1. Descriptive statistics of the milk performance of Holstein and Czech Simmental cows

	1st lactation					2nd lactation				
Trait	n	Mean	SD	Min.	Max.	n	Mean	SD	Min.	Max.
Milk yield (kg)	170	8,222	1,881	4,294	13,285	156	9,125	2,178	5,245	1,5453
Milk fat (%)	170	4.16	0.32	3.41	5.28	156	4.17	0.37	3.36	5.31
Milk fat (kg)	170	340.9	76.4	182.0	573.0	156	379.0	90.8	206.0	617.0
Crude protein (%)	170	3.46	0.22	2.85	3.94	156	3.50	0.22	2.95	4.01
Crude protein (kg)	170	281.5	53.5	166.0	430.0	156	316.2	65.2	201.0	489.0

n number of lactations of cows; SD standard deviation.

Table S2. Descriptive statistics of the milk quality of Holstein and Czech Simmental cows

	1st and 2nd lactations				
	n	Mean	SD	Min.	Max.
Milk fermentation ability (ml NaOH)	335	14.85	4.07	6.73	35.5
Renneting subjectively (sec)	363	491.41	216.263	188	1600
Renneting instrumentally (sec)	338	303.85	128.62	20	781
Ethanol test (ml of ethanol)	343	0.994	1.011	0.20	5.50
Somatic cell score	355	2.862	1.567	0.356	8.761
Lactose monohydrate (%)	355	4.93	0.22	3.65	5.58
Citric acid (%)	354	0.19	0.03	0.11	0.34
Acetone (mmol/L)	354	0.14	0.11	0.01	0.94
Ketones BHB[1] (mmol/L)	351	0.10	0.17	0.01	1.46
Urea (mg/100 ml)	355	27.2	6.6	8.1	55.4
Not fat solid (%)	272	9.08	0.42	7.75	10.23
Casein (%)	151	2.79	0.34	2.02	3.83

n number of repeating measuring of samples; SD standard deviation; [1]betahydroxybutyrate.
